# Evaluating the Efficacy of 0.5% Sodium Hypochlorite Disinfection for Microbial Control in Korle Bu Teaching Hospital's Burns and Plastics Unit

**DOI:** 10.4314/ejhs.v35i2.6

**Published:** 2025-03

**Authors:** Collins Amponsah, Emmanuel U Osisiogu, Mark Addy, Philip Asumang, Frank Kwasikumah, Enid Owusu

**Affiliations:** 1 Department of Medical Laboratory Sciences, School of Biomedical and Allied Health Sciences, University of Ghana, Legon; 2 Department of Science Laboratory Technology, Dr. Hilla Limann Technical University, Wa, Ghana; 3 Department of Science, Seventh-Day Adventist College of Education, Agona – Ashanti, Ghana; 4 Department of Medical laboratory, Danpong Medical Centre, Accra, Ghana

**Keywords:** Sodium hypochlorite, Minimum Inhibitory Concentration, Minimum Bactericidal Concentration, Nosocomial infections, Efficacy, Disinfectants

## Abstract

**Background:**

The rise in nosocomial infections, also known as healthcare-associated infections (HAIs), has led to widespread illness and fatalities, affecting both patients and healthcare workers. This surge is a result of inadequate disinfection practices. We aimed to evaluate the efficacy of a 5% disinfectant on microbial contamination in the Burns Unit of Korle-Bu Teaching Hospital (KBTH).

**Methods:**

Swab samples were collected from surfaces such as working benches, door handles, sinks, taps, and trolleys in the Burns Unit of KBTH before and after routine disinfection. The samples were cultured on Blood agar and MacConkey agar. Standard bacteriological techniques, including Gram staining and biochemical tests, were used to identify the isolated bacteria. The isolates were then tested against prepared dilutions of the bleach disinfectant used in the Burns Unit. Additionally, the Minimum Inhibitory Concentration (MIC) and Minimum Bactericidal Concentration (MBC) of sodium hypochlorite against the isolates were determined.

**Results:**

Statistical analysis revealed no significant difference in microbial load before and after routine disinfection for most sampled surfaces (p > 0.05), except for air-exposed plates. The isolated organisms included Staphylococcus aureus, coagulasenegative staphylococci, Streptococcus spp., Pseudomonas spp., Klebsiella pneumoniae, and Citrobacter freundii. While the working solution showed no inhibition zones, laboratory testing confirmed that a properly prepared 0.5% sodium hypochlorite solution was effective against all isolates.

**Conclusion:**

This study found that the bioburden remained high after routine disinfection. A 0.5% sodium hypochlorite solution (1:10 dilution of the 5% stock) was effective in eliminating all isolates.

## Introduction

Hospital-acquired infections (HAIs) present a serious public health challenge, particularly in developing countries where healthcare resources are limited. In such settings, overcrowded hospitals, poor sanitation, and inadequate access to clean water and soap are prevalent, leading to increased infection rates. Burns and plastic surgery patients are especially vulnerable to HAIs due to their weakened immune systems and extensive wounds (1). These procedures often involve breaking the skin, providing entry points for bacteria and other microorganisms (2). Moreover, prolonged hospital stays elevate the risk of exposure to HAIs.

The high incidence of HAIs in the Burns and Plastics Department of Korle Bu Teaching Hospital (KBTH), Ghana's largest hospital, is a major concern (3). Despite various infection control measures, such as hand hygiene protocols and environmental cleaning, HAIs remain a persistent issue, highlighting the need for more effective strategies to reduce microbial load.

Hand hygiene protocols are essential for preventing the spread of HAIs, yet compliance among healthcare workers is often inconsistent, especially in developing countries (4). While environmental cleaning plays a role in reducing microbial load, thoroughly disinfecting all surfaces in a hospital is a challenging task. Disinfectants have proven effective in lowering microbial contamination on surfaces and equipment in healthcare settings. Studies have shown that low compliance with disinfection protocols in U.S. burn units was linked to a higher risk of HAIs (5). Additionally, hydrogen peroxide fogging has been shown to significantly reduce microbial contamination in patient rooms, yielding a 98% improvement in aerobic colony counts (6). Alcohol-based hand rubs have also been effective in curbing bacterial transmission in healthcare settings (7, 8).

While many studies have examined the effectiveness of disinfection in developed countries, research on disinfectant efficacy in developing countries is limited. Therefore, this study aimed to evaluate the effectiveness of 5% sodium hypochlorite in reducing microbial load at KBTH's Burns and Plastics Department. We hypothesized that routine disinfection with 5% sodium hypochlorite would significantly reduce surface microbial contamination. The findings aim to provide valuable insights into disinfectant effectiveness and help improve patient outcomes in resource-limited settings.

## Methods

This study utilized a prospective experimental design, and the treatment room in the Burns and Plastics Department of KBTH was selected as the study area.

**Sample collection**: Sterile cotton swabs soaked in normal saline were used to swab procedure benches, sinks, door handles, trolleys, and taps within the treatment rooms of the burn unit before disinfection. Three swabs were collected from each surface. Blood agar and MacConkey agar plates were exposed to air for 30 minutes before being incubated at 37°C overnight. The routine disinfection of the area was performed by hospital staff using Powerzone (5% sodium hypochlorite) as the standard disinfectant. After a 30-minute contact time, the sampling process was repeated. This postdisinfection sampling followed the same procedure as the pre-disinfection sampling, including the 30-minute exposure of Blood agar and MacConkey agar plates. Proper labeling was ensured for all samples, which were transported to the microbiology laboratory for analysis. The procedures were repeated four times on different days to account for real-world variation in disinfection practices.

**Sample processing and bacterial identification**: Bacterial enumeration was performed by eluting each swab in 10 mL of sterile saline. The samples were then subjected to double dilution, and the diluted samples were applied to labeled plate count agar (PCA) and blood agar (BA). The plates were incubated at 37°C for 24 hours, and colony enumeration was carried out manually. Gram staining and biochemical tests (catalase, coagulase, oxidase, triple sugar iron, urease, and indole tests) were conducted to identify bacterial isolates.

**MINIBACT-E technique**: For Gram-negative rods, pure colonies were dispersed in 0.2 mL of peptone water and compared to a 1% McFarland standard. The MINIBACT-E cassette, which contains 96 wells with reagents for specific biochemical parameters, was used to identify bacteria by color change in the wells. The strain numerical codes were compared with a database for bacterial identification.

**Antimicrobial susceptibility testing**: The Kirby-Bauer method was used to test the antimicrobial susceptibility of bacterial isolates against sodium hypochlorite. The diameter of inhibition zones was measured and compared with standard reference values from the Clinical and Laboratory Standards Institute (12).

**Quality control of 0.5% Sodium Hypochlorite**: Isolates *Staphylococcus aureus*, *Pseudomonas spp.*, and *Klebsiella pneumoniae* were selected for testing based on their clinical relevance and resistance profiles. An inoculum of each organism was prepared, and the antimicrobial susceptibility of sodium hypochlorite was tested using the Kirby-Bauer method.

**Determination of minimum inhibitory concentration (MIC) and minimum bactericidal concentration (MBC)**: The MIC and MBC of sodium hypochlorite against the isolates were determined using the broth dilution method. Twelve falcon tubes were prepared with sterile distilled water, and serial dilutions of sodium hypochlorite were performed. Tubes were incubated at 37°C for 24 hours, and the MIC was defined as the lowest concentration preventing visible bacterial growth. For MBC determination, tubes with no visible growth were sub-cultured onto Mueller Hinton Agar plates, and the MBC was defined as the lowest concentration with no bacterial growth on the plate.

**Data analysis and management**: Data were input into Microsoft Excel and analyzed using SPSS version 20.0. A paired t-test was used to assess the significant variation in microbial load before and after disinfection. Results were presented as means with standard deviation, and p-values ≤ 0.05 were considered statistically significant.

## Results

**Figure 1:**
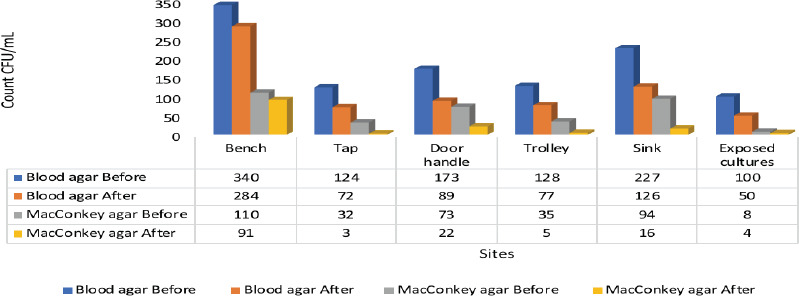
A bar chart showing the mean colony count on both Blood Agar and MacConkey agar before and after disinfection and the sites of sample collection

**Colony count and statistical analysis**: Microbial counts were determined using the spread plate method before and after disinfection (Figure 1), and the data were analyzed using paired t-tests (Table 1). Results showed no significant difference in colony counts before and after disinfection for most sites (p > 0.05), except for air-exposed plates, where a significant difference was observed (p < 0.05).

**Table 1:** Paired t-test comparing the plate count before and after disinfection for the various sites sampled

Site	Batch 1		Batch 2		Batch 3		Batch 4		Mean ± SD		p-value

	Before	After	Before	After	Before	After	Before	After	Before	After	
Bench	500	312	92	33	284	144	484	376	340±192	216±157	0.113
Tap	63	32	44	32	5	2	384	224	124±175	72±102	0.254
Door handle	81	55	8	6	16	10	588	286	173±278	89±133	0.332
Trolley	63	38	220	108	144	84	84	76	128±70	77±29	0.112
Sink	143	110	8	3	240	121	420	236	227±169	126±96	0.072
Plate exposed to air	110	66	100	32	156	88	32	16	100±51	50±33	0.029

Microbiological analysis identified several bacterial species including *Staphylococcus aureus* (found at all sampled sites), *Streptococcus* species, coagulase-negative *Staphylococci*, *Pseudomonas species*, *Klebsiella pneumoniae*, and *Citrobacter freundii* (Tables 2 and 3). The MINIBACT-E kit was used to identify lactose-fermenting bacteria.

**Table 2:** Species of PPIAs isolated

ISOLATE	SITE	BIOCHEMICAL TEST			

		GRAM	CATALASE	COAGULASE	OXIDASE
*Staphylococcus aureus*	Bench	GPC	Positive	Positive	
	Door handle				
	Sink				
	Open				
	Trolley				
	Tap				
*Streptococcus spp*	Bench	GPC	Negative	Negative	
	Door handle				
	Sink				
	Open				
	Trolley				
*Coagulase negative* *Staphylococcus*	Bench	GPC	Positive	Negative	
	Open				
	Sink				
	Tap				
*Pseudomonas spp*	Bench	GNR			Positive
	Door handle				
	Sink				
	Trolley				
*Klebsiella pneumoniae*	Bench	GNR			Negative
	Sink				
	Door handle				
	Trolley				
*Citrobacter freundii*	Open	GNR			Negative
	Bench				

KEY: GPC = Gram Positive Cocci

GNR=Gram Negative Rod

**Table 3:** Enterobacteria identification with MINIBACT-E

	Positive reaction codes	4	2	1	4	2	1	4	2	1	4	2	1	4	2	1	4		
Lactose	Respondent/Isolate ID	H^2^S	NO^3^	PD	INDOLE	LYSINE	OD	MAL	UREA	VP	ESC	ONPG	SAK	ARAB	ADON	INOS	SORB	Strain numerical code	Strain ID
		-	+	-	-	+	-	+	+	-	+	+	+	+	+	+	+	226774	*Klebsiella pneumoniae*
		-	+	-	-	-	+	+	-	-	-	+	+	+	-	-	+	214344	*Citrobacter freundii*

KEY: H_2_S – Hydrogen sulphide, ONPG – β-galactosidase, NO^3^ – Nitrate, SAK – Sucrose, PD – Phenylalanine, ARAB – Arabinose, LD – Lysine, ADON – Adonitol, OD – Ornithine, INOS – Inositol, MAL – Malonate, SORB – Sorbitol, VP – Voges Proskauer, ESC – Esculin

**Susceptibility testing of stock and working solution**: The stock sodium hypochlorite (5%) and the working solution (0.5%) were tested against three bacterial isolates (*Staphylococcus aureus*, *Pseudomonas spp.*, and *Klebsiella pneumoniae*). The working solution showed no zone of inhibition, indicating it was ineffective under the tested conditions.

**Minimum inhibitory concentration (MIC) and minimum bactericidal concentration (MBC)**: The MIC values of sodium hypochlorite were recorded as 0.00005% for all isolates (Table 5), with varying MBCs. *Klebsiella pneumoniae* and the pooled isolates exhibited the highest MBC (0.05%), while *Citrobacter freundii* had the lowest MBC (0.005%).

**Table 5:** MICs and MBCs of isolates in percentage

ISOLATE	MIC, %	MBC, %
*Staphylococcus aureus*	5×10^-5^	0.050
*Pseudomonas spp*	5×10^-5^	0.050
*Klebsiella pneumoniae*	5×10^-5^	0.500
*Coagulase negative Staphylococcus*	5×10^-5^	0.050
*Streptococcus spp*	5×10^-5^	0.050
*Citrobacter freundii*	5×10^-5^	0.005
Pooled isolates	5×10^-5^	0.500

## Discussion

This study aimed to assess the effectiveness of 0.5% sodium hypochlorite in reducing microbial contamination in the Burns and Plastics Department of KBTH. The results showed no significant difference in microbial load before and after disinfection, suggesting that routine disinfection was not effective. The bioburden levels on Blood Agar (BA) samples collected from frequently touched surfaces like the bench and door handle showed no significant difference before and after disinfection. These areas, commonly contacted by both patients and healthcare workers, may require more frequent or thorough disinfection protocols. Mouajou et al. (2022) highlighted that hand hygiene compliance varies significantly among healthcare workers, particularly in resourcelimited settings, which could explain the persistent contamination on these surfaces (4). A previous study has identified healthcare workers' hands as a primary reservoir for transmitting Klebsiella spp. (14), which could explain the high bioburden observed on the door handle before and after disinfection. The 0.5% sodium hypochlorite solution demonstrated effectiveness against all isolates in laboratory tests, indicating that the disinfectant could be useful in controlling microbial contamination if properly prepared and applied.

The hospital protocol specifies the preparation of a 1:10 (0.5%) dilution from 5% stock sodium hypochlorite for routine disinfection in the burns unit. However, testing this dilution against three isolates via the Kirby-Bauer method showed no zone of inhibition. This discrepancy suggests that the working solution used might not be a true 1:10 dilution as claimed. A previous study emphasized that dilute sodium hypochlorite has a shelf life of just 24 hours compared to the stock's six months [6], which could explain our observations if solutions were prepared improperly or used beyond their effective period.

In conclusion, while no significant reduction in microbial load was observed in real-world settings, laboratory testing confirmed the efficacy of a 0.5% sodium hypochlorite solution against various microorganisms. The study emphasizes the importance of proper disinfection practices and the need for consistent preparation and application of disinfectants in healthcare settings to prevent HAIs.

## Figures and Tables

**Table 4:** Test isolates and their zones of inhibition

Isolate	Zone of inhibition, mm
*Staphylococcus aureus*	31
*Pseudomonas spp*	34
*Klebsiella pneumoniae*	29
